# Naturally derived bioactive compounds as regulators of oxidative stress and inflammation in asthma

**DOI:** 10.1186/s13020-025-01142-w

**Published:** 2025-07-01

**Authors:** Jangho Lee, Hyo-Kyoung Choi, Hee Soon Shin, Gun-Dong Kim

**Affiliations:** 1 Division of Food Functionality Research, Korea Food Research Institute (KFRI), Wanju, 55365 Republic of Korea; 2 Department of Food Biotechnology, Korea University of Science and Technology (UST), Daejeon, 34113 Republic of Korea

**Keywords:** Asthma, Allergic respiratory disease, Oxidative stress, Reactive oxygen species, Inflammation

## Abstract

Asthma is a chronic allergic respiratory disease characterized by symptoms such as coughing, dyspnea, and reversible airway obstruction. The incidence of asthma has been gradually increasing worldwide. However, the pathophysiological mechanisms underlying its development remain unclear due to its multifactorial etiology, which encompasses genetic, environmental, and occupational factors. Furthermore, the clinical manifestations of asthma vary significantly among individuals and across age groups, often coexisting with symptoms of atopic dermatitis and allergic rhinitis, thereby necessitating a personalized and continuous therapeutic approach. Asthma management primarily involves the use of symptom relievers and anti-inflammatory controllers, including β₂-agonists, anticholinergics, and corticosteroids. However, prolonged or high-dose administration of these agents poses a risk of adverse effects. Given these limitations, the development of novel asthma therapies with enhanced efficacy and fewer side effects requires a deeper understanding of the pathophysiological mechanisms underlying the development of the disease. Existing evidence from various preclinical studies suggests that oxidative stress and inflammatory responses play pivotal roles in the onset and exacerbation of asthma. Therefore, the purpose of this review was to address the multifaceted pathological mechanisms of asthma, highlight naturally derived bioactive compounds with potential antioxidative and anti-inflammatory properties that could be beneficial for asthma management. Additionally, propose an integrative therapeutic strategy that enhances patient adherence while minimizing adverse effects, ultimately contributing to improved long-term management and treatment of asthma.

## Background

Asthma is a chronic allergic respiratory disease characterized by reversible limitation of expiratory airflow due to airway inflammation, bronchoconstriction, and excessive mucus secretion [1]. The primary symptoms of asthma include dyspnea, wheezing, and cough, which result from airway narrowing caused by excessive contraction of bronchial smooth muscles, edema, and increased airway hyperresponsiveness (AHR) [2]. The global prevalence of asthma has been steadily increasing, with over 262.4 million cases reported worldwide in 2019. In addition, the estimated prevalence of asthma is projected to reach 400 million by 2025. Inadequate asthma management and exacerbations can lead to mortality. Specifically, approximately 461.1 thousand asthma-related deaths were reported worldwide in 2019 [3]. Asthma is a resource-intensive chronic disease with a high prevalence and a significant risk of acute exacerbations impairs quality of life, reduces physical function, and increase economic burden on individuals and society. Regarding disability-adjusted life years (DALYs), asthma accounted for 20% (approximately 21.6 million DALYs) of the total burden of chronic respiratory diseases in 2019, ranking 34th among all diseases [3, 4].

Asthma is a heterogeneous disease with diverse phenotypic presentations that is influenced by various host factors, including genetic predisposition, obesity, and sex, as well as environmental factors, such as allergens, infections, and air pollution [5]. Genetic factors play a pivotal role in the pathogenesis of asthma, with reports indicating that 25–80% of patients with asthma have a family history of the disease [6]. Recent genome-wide association studies of genetic variants have demonstrated that multiple genetic polymorphisms contribute to asthma susceptibility and phenotypic heterogeneity [7, 8]. Obesity is associated with a 1.9–2.3-fold increased risk of asthma compared to normal weight. In addition, elevated levels of leptin derived from adipose tissue have been implicated in airway inflammation and heightened AHR [9–11]. Furthermore, the prevalence of asthma is higher in males than in females before the age of 14; however, females exhibit a higher prevalence in adulthood [12]. Regarding environmental factors, the associations between smoking and air pollution and the pathogenesis of asthma have been studied extensively. In patients with asthma, smoking accelerates the decline in lung function, exacerbates symptoms, and reduces the efficacy of both inhaled and systemic corticosteroid therapy, leading to poor asthma control [13, 14]. Moreover, prenatal and postnatal exposure to smoking increases the risk of asthma-like symptoms in children by 21–85%. In addition, infants born to mothers who smoked during their pregnancy have a fourfold increased likelihood of developing wheezing within the first year of life [15, 16]. Indoor and outdoor air pollution have been recognized as significant etiological factors for asthma. Previous reports have indicated that ozone, nitrogen dioxide, and particulate matter (PM) are strongly associated with the development of asthma. Epidemiological studies have demonstrated long-term exposure to PM2.5 and PM10 increases the risk of asthma in adults [17–19]. In addition, a prospective birth cohort study conducted in the Netherlands demonstrated that elevated PM2.5 concentrations are associated with an increased risk of wheezing (odds ratio [OR], 1.2; 95% confidence interval [CI], 1.0–1.4) and physician-diagnosed asthma (OR, 1.3; 95% CI, 1.0–1.7). Furthermore, evidence from some studies have indicated that PM2.5 exposure is correlated with increased incidence (OR, 1.28; 95% CI, 1.10–1.49) and prevalence (OR, 1.26; 95% CI, 1.04–1.51) of asthma [20, 21].

Asthma has a highly complex pathogenesis. The pathological process of the development of asthma is mediated by various immune cells and inflammatory mediators, with the T helper (Th) 2 cells playing crucial roles in eosinophilic inflammation, immunoglobulin E (IgE) production by B lymphocytes, and reduction of regulatory cells associated with immune tolerance [22]. Upon allergen exposure, epithelial cells release growth factors that activate fibroblasts, leading to excessive collagen deposition and subsequent airway smooth muscle hypertrophy [23]. Additionally, inflammatory mediators are released, facilitating dendritic cell activation in response to specific antigen recognition [24]. In the airway, activated immune cells utilize antigen presentation to secrete cytokines such as interleukin (IL) 4, IL5, IL9, and IL13, which promote eosinophilic inflammation and induce IgE production [25]. IgE binds to mast cells, triggering the release of histamine and cysteinyl leukotrienes, which contribute to bronchoconstriction, edema, and mucus hypersecretion [26]. Dysregulated immune responses disrupt immune tolerance, playing a crucial role in the development and exacerbation of chronic allergic asthma. In severe asthma, type 2 innate lymphoid cells, which are pivotal in the production of IL4, IL5, and IL13, as well as in T lymphocyte regulation, are markedly increased, along with macrophages, neutrophils, Th1, and Th17 cells, contributing to disease progression [27–29]. These inflammatory responses affect epithelial cells, leading to airway AHR, reversible airflow limitation, and airway remodeling [30].

The treatment of asthma generally involves a combination of therapies, such as inhaled corticosteroids, anticholinergics, short-acting β-agonists, and long-acting β-agonists [1, 31]. However, the efficacy of these therapies tends to decrease without consistent and personalized treatment and management tailored to disease duration and clinical symptoms. Moreover, steroid resistance has been reported in 10–15% of patients with asthma. In addition, prolonged use of systemic corticosteroids can lead to hypothalamic–pituitary–adrenal axis dysfunction [32, 33]. Asthma is often accompanied by comorbid conditions such as allergic rhinitis, sinusitis, and nasal polyps, which can contribute to increased hospitalization rates [34–37]. Although biological therapies have emerged as targeted treatment options for asthma, they impose a significant economic burden and pose potential risks of adverse effects. Therefore, further research is needed to elucidate the pathophysiology of asthma and optimize the existing treatment strategies.

This review aimed to enhance our understanding of oxidative stress and inflammatory responses, which are major contributors to the pathophysiology of asthma, by examining the various cells and mediators involved in these processes. Additionally, we explore the diverse oxidative stress- and inflammation-related signaling pathways implicated in the pathogenesis and exacerbation of asthma. Furthermore, we discuss a multifaceted approach to developing alternative therapeutics that can overcome the limitations of the existing biological agents used in asthma management. Specifically, we highlight naturally derived bioactive compounds with antioxidant and anti-inflammatory properties and examine their potential applications in preclinical asthma models for the development of sustained and patient-compliant therapeutic strategies with fewer adverse effects.

## Pathophysiology of asthma

**Fig. 1:**
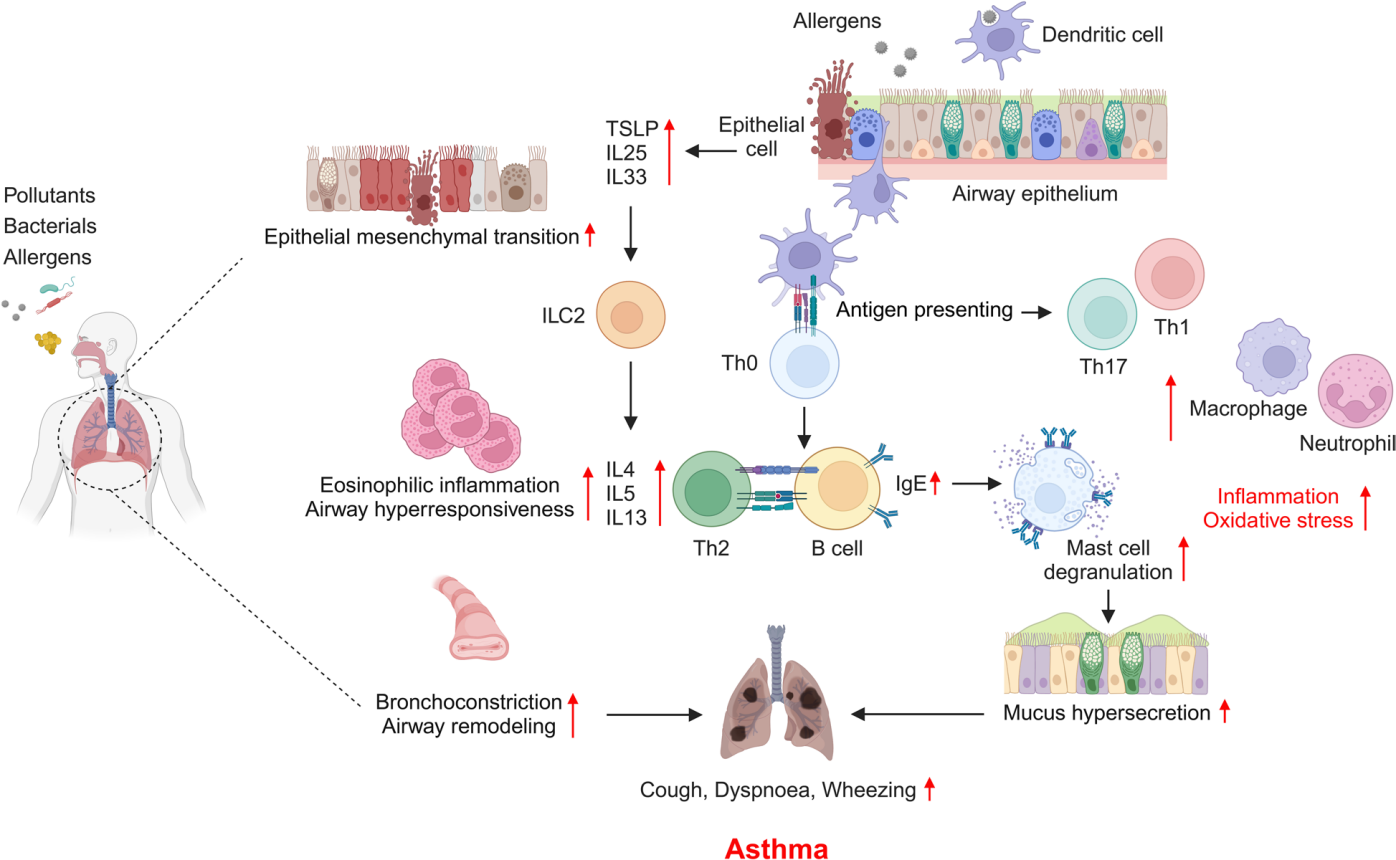
Pathogenesis of asthma. Asthma is caused and exacerbated by repeated exposure to various harmful substances, including environmental pollutants, bacteria, microbes, and allergens. In addition, asthma is characterized by hypersensitivity and dysregulated immune responses mediated by pro-inflammatory mediators and activated immune cells, such as dendritic cells, eosinophils, mast cells, neutrophils, macrophages, and lymphocytes. Asthma becomes chronic and worsens as it progresses, leading to airway hyperresponsiveness and airway remodeling. Immunoglobulin E, IgE; interleukin, IL; type 2 innate lymphoid cell, ILC2; T helper, Th; thymic stromal lymphopoietin, TSLP. Created with BioRender.com. Accessed on 17 March 2025

The pathological changes that occur in asthma involve complex interactions between structural and inflammatory alterations in the airways (Fig. 1). These mechanisms manifest as several distinct but interconnected pathophysiological features that contribute to the clinical presentations of the disease. The subsequent sections describe four key pathophysiological components of asthma: persistent airflow limitation, airway hyperresponsiveness, airway inflammation, and hyperproduction of airway mucus. Each component represents specific cellular and molecular events that not only characterize the disease process but may also be potential targets for therapeutic intervention. Recent advances in understanding these mechanisms have revealed important similarities and differences between severe asthma and other chronic respiratory conditions, particularly in relation to tissue remodeling and inflammatory cascades.

### Airflow limitation

Chronic airflow limitation in asthma is characterized by increased airway resistance and decreased lung compliance, often becoming irreversible over time. The development of this condition is influenced by various factors, including history of asthma exacerbation, poor adherence to treatment, and comorbidities such as obesity and pneumonia, which are significant risk factors for persistent airflow limitation in children [38]. When asthma is suspected, spirometry should be performed according to the criteria established by the American Thoracic Society to assess the presence of airway reversibility and limitation. Typically, forced expiratory volume in one second (FEV₁) and forced vital capacity (FVC) are measured to calculate the FEV₁/FVC ratio, with an FEV₁/FVC ratio of < 0.75–0.8 considered indicative of expiratory airflow limitation [39, 40]. In adults, chronic airflow limitation is more prevalent in those with late-onset asthma, with age being a significant factor that influences its development [41]. The pathophysiology of severe asthma with persistent airflow limitation shares similar features with that of chronic obstructive pulmonary disease (COPD), including airway remodeling and emphysema-like changes, which complicate diagnosis and treatment [42]. Additionally, clinically relevant early life events, such as lower respiratory tract infections, can predispose individuals to fixed airflow limitation later in life. Therefore, early intervention is essential for improved outcomes in adulthood [43]. On a cellular level, increased levels of IL25R^+^ circulating fibrocytes are associated with severe asthma and correlated with fixed airflow limitation, indicating a potential target for therapeutic intervention [44].

### Airway hyperresponsiveness

AHR, a key clinical feature of asthma, is characterized by excessive airway constriction in response to stimuli that do not elicit reactions in healthy individuals. AHR is known to trigger airway inflammation, recurrent wheezing, dyspnea, and symptoms associated with asthma severity in response to various stimuli [45]. The mechanisms underlying AHR can be broadly categorized as follows: (1) hypertrophy of airway smooth muscle cells and increased contractility [46], (2) loss of the maximal plateau of contraction observed in normal bronchi upon inhalation of bronchoconstrictive agents due to airway inflammation [47], (3) thickening of the airway wall due to edema and structural remodeling, leading to airway smooth muscle contraction [48], and (4) sensitization of sensory nerves by inflammatory processes [49]. AHR can be classified into direct and indirect types based on the mechanism of airway reactivity. Direct AHR refers to heightened bronchoconstriction in response to inhaled pharmacological agents such as histamine and methacholine, which directly stimulate airway smooth muscle receptors [50, 51]. Indirect AHR is characterized by increased sensitivity to endogenous mediators released by activated airway cells, such as mast cells [52].

Of the various inflammatory cells, mast cells infiltrating airway smooth muscle are particularly implicated in AHR. These cells interact with Th2 cells to produce a range of lipid mediators, chemokines, cytokines, and enzymes, inducing structural changes in the airway that contribute to persistent AHR [53, 54]. AHR may also manifest transiently depending on the patient’s inflammatory state. Overexpression of inflammatory genes by mast cells and eosinophils within the airway epithelium has been associated with increased epithelial inflammation and indirect AHR in patients with asthma undergoing inhaled corticosteroid therapy [55, 56]. Recent studies have demonstrated that damaged airway epithelium plays a critical role in mediating inflammation-driven indirect AHR in asthma through the release of cytokines such as thymic stromal lymphopoietin and IL33 [57, 58]. Several studies have demonstrated that AHR is not exclusive to patients with asthma. In the SAPALDIA cohort studies, approximately 8% of individuals without any respiratory symptoms exhibited AHR. The results of the study indicated that these individuals had a more than fourfold increased risk of developing asthma compared to those without AHR, suggesting that AHR is an important risk factor for the development of airway disease [59]. Furthermore, the degree of AHR is positively correlated with asthma severity. A recent study indicated that concurrent exposure to indirect triggers, such as viral infections, allergens, occupational irritants, and exercise, can temporarily exacerbate AHR [60–62].

### Airway inflammation

Chronic airway inflammation is a common feature of allergic asthma. Activation of mast cells leads to enhanced secretion of inflammatory and bronchoconstrictive mediators, such as histamine, cysteinyl leukotrienes, and prostaglandin D2, as well as increase in the number of activated eosinophils [1]. Eosinophils release major basic protein, which damages the airway epithelium and contributes to airway remodeling [63]. Increasing numbers of Th1, Th2, and Th17 cells promote the production of cytokines such as IL4, IL5, IL9, and IL13, which further drive eosinophilic inflammation and stimulate B lymphocytes to produce IgE, thereby amplifying the allergic inflammatory response [25, 64]. In addition, increased numbers of neutrophils and macrophages activated by allergens bound to low-affinity IgE receptors lead to elevated production of inflammatory mediators, cytokines, and matrix metalloproteinases [65, 66]. Consistent with these observations, a recent study showed that a large number of neutrophils and macrophages infiltrate the airways and sputum of patients with severe asthma and those with a history of smoking [67]. Innate type 2 lymphoid cells (ILC2), which are regulated by epithelial-derived inflammatory mediators such as IL25 and IL33, also contribute to asthma-related inflammation [68]. Neutrophils are also involved in severe asthma [69]. Recent studies have identified ILC2 as a key driver of eosinophilic airway inflammation in both allergic and non-allergic asthma [26]. Beyond inflammatory cells, structural cells within the airway also generate inflammatory mediators and sustain inflammation through diverse mechanisms, further perpetuating the chronic inflammatory response in asthma.

### Hyperproduction of airway mucus

Excessive mucus secretion, a hallmark of chronic bronchitis and asthma, is driven by complex cellular and molecular mechanisms that involve goblet cell hyperplasia and hypertrophy of mucus-secreting glands. In asthma, the pathogenesis of excessive mucus production is primarily attributed to increased numbers of goblet cells, which are stimulated by inflammatory mediators such as leukotrienes, proteolytic enzymes, and neuropeptides [70, 71]. These mediators activate signaling pathways that lead to the differentiation and proliferation of goblet cells, resulting in increased mucin production, the gel-forming component of mucus [70]. Mucus hyperconcentration is a critical factor in chronic bronchitis, in which abnormalities in mucus hydration and concentration impede mucociliary clearance, exacerbating airflow limitation [72]. The role of neutrophils in both asthma and chronic bronchitis is significant because they release serine proteases that potentiate mucin secretion and contribute to the inflammatory milieu [73]. Additionally, cigarette smoke and microbial products are potent inducers of mucin production, which further complicates the pathophysiology of COPD in smokers [73, 74]. Excessive mucus not only obstructs airways but also serves as a medium for bacterial growth, propagating inflammation and disease progression [75]. Some pharmacotherapies, such as corticosteroids and mucolytics, target mucus hypersecretion; however, their efficacies vary, particularly in patients with COPD compared to those with asthma [76]. Novel therapeutic strategies focused on inhibition of mucin synthesis, secretion, and goblet cell hyperplasia are being explored, with the goal of offering more targeted and effective treatments for managing mucus hypersecretion in respiratory diseases [76].

## Etiology of asthma

**Fig. 2:**
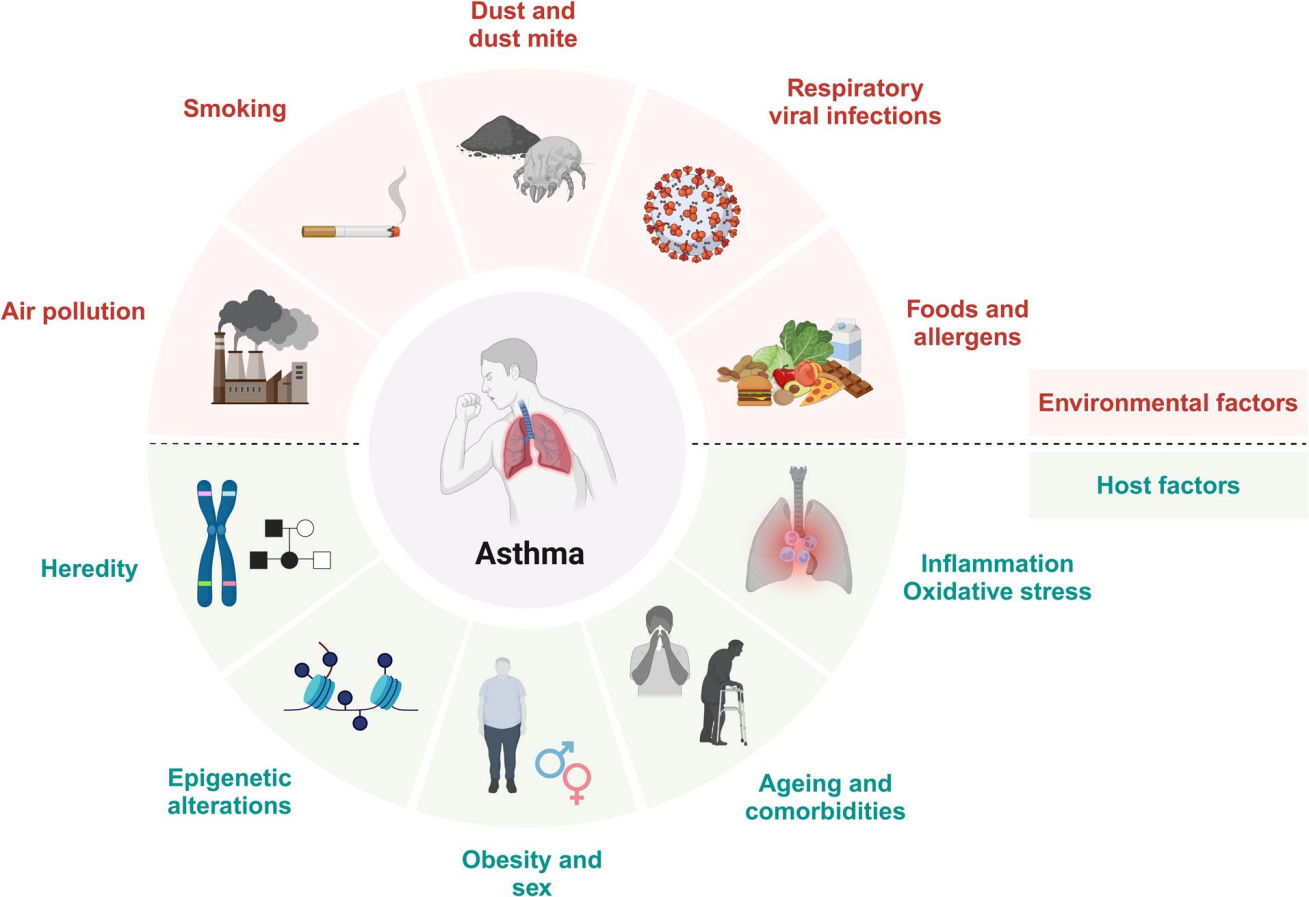
Etiology of asthma. Asthma is a chronic allergic respiratory disease with a highly complex and multifactorial etiology. The pathogenesis of asthma is broadly classified into host factors and environmental factors. The host factors include family history, genetic and epigenetic modifications, obesity, sex, aging, comorbidities, inflammation, and oxidative stress. The environmental factors encompass air pollution, smoking, viral infections, various allergens, and psychological stress. A comprehensive understanding of the interactions between pathogenic mechanisms is essential for the prevention, management, and treatment of asthma. Created with BioRender.com. Accessed on 17 March 2025

The etiology of asthma encompasses several interconnected pathophysiological mechanisms that contribute to airway inflammation and bronchial hyperresponsiveness (Fig. 2). The following sections highlight the key factors that drive the development and progression of asthma, beginning with oxidative stress as a fundamental process that initiates and amplifies inflammatory cascades in the airways.

### Oxidative stress

Oxidative stress plays a significant role in the etiology of asthma, primarily through the imbalance between the production of reactive oxygen species (ROS) and the antioxidant defense system, leading to cellular damage and inflammation. This imbalance is exacerbated by environmental factors such as cigarette smoke, allergens, viruses, and pollutants such as PM2.5, which increase ROS levels and contribute to airway inflammation and hyperresponsiveness [77, 78]. The presence of oxidative stress in asthma is linked to the activation of inflammatory cells and mediators, which further stimulate inflammation and airway obstruction [79]. Oxidative stress is a determinant of asthma onset and progression in children, with lifestyle factors and environmental exposures playing crucial roles [78]. The antioxidant system, both endogenous and exogenous, is vital for counteracting oxidative stress, and its dysfunction can lead to bronchial remodeling and increased asthma severity [79]. Despite the known effects of oxidative stress on asthma, the efficacy of therapeutic strategies targeting oxidative burden has been largely disappointing. This highlights the complexity of oxidative pathways and the need for effective antioxidant therapies [80]. Moreover, oxidative stress contributes to corticosteroid insensitivity in cases of severe asthma, disrupting glucocorticoid receptor signaling and sustaining pro-inflammatory pathways. This underscores the importance of optimizing antioxidant responses to enhance the efficacy of corticosteroids [81]. In obese individuals, oxidative stress, along with dysbiosis, may play a key role in asthma pathogenesis. However, dietary interventions and probiotics have shown potential in reducing the levels of oxidative stress markers and improving antioxidant capacity [82]. Overall, maintaining a balance between oxidant and antioxidant systems is crucial for managing asthma and preventing its progression. However, further research is needed to develop effective antioxidant-based therapies for asthma.

### Inflammatory mediators

Various inflammatory mediators recruit inflammatory cells to inflammatory lesions or vasculature and trigger inflammatory responses, leading to structural changes in the lungs. Patients with asthma exhibit elevated serum levels of cytokines such as IL6, IL8, TNFα, and IL1β, which activate immune cells including neutrophils, macrophages, and lymphocytes, contributing to the initiation and development of inflammatory responses [83, 84]. Neutrophils in particular play significant roles in the pathogenesis of inflammatory airway diseases, including asthma, due to their unique functions and retention in the lung vasculature. Neutrophils release inflammatory mediators such as neutrophil elastase and neutrophil extracellular traps, which can dramatically affect disease outcomes [85]. The inflammatory response involves a complex interplay between leukocytes, including macrophages and lymphocytes, which release substances such as vasoactive amines, eicosanoids, and proinflammatory cytokines to mediate inflammation and promote tissue healing [86]. The recruitment of leukocytes to the lungs is a hallmark of lung inflammation, often triggered by environmental factors and mediated by cytokines and chemokines [87]. In asthma, the inflammatory process is further complicated by the involvement of various effector cells, including eosinophils, basophils, and mast cells, which contribute to increased vascular permeability, mucus secretion, and structural changes in airway architecture [88].

### Inflammatory cells

In asthma, dendritic cells (DCs) play a crucial role in initiating immune responses to inhaled allergens within the airways [89]. Izumi et al. conducted a single-cell RNA sequencing (scRNA-seq) analysis of lung tissues from mice exposed to house dust extract and identified distinct subsets of CD11b⁺ DCs, including Ly-6C⁺, CD301b⁺, and CD200⁺ populations. The authors observed that mature CD11b⁺ DC subsets promoted the differentiation of T lymphocytes into Th2 and Th17 cells [90]. Neutrophils are known to exacerbate the severity of asthma and reduce responsiveness to corticosteroid treatment [91]. In a recent study conducted using mouse models of house dust mite (HDM)-induced asthma, deficiency of programmed cell death protein 1, which is highly expressed in pulmonary T lymphocytes, was associated with acute neutrophilic AHR [92]. During respiratory inflammation, the number of activated macrophages in the peripheral airways, pulmonary parenchyma, and pulmonary vasculature increases. This increase in activated macrophages is accompanied by recruitment of activated neutrophils and lymphocytes, such as cytotoxic T cell type 1, Th1, and Th17 cells. Macrophages, particularly alveolar macrophages, play a pivotal role in orchestrating this inflammatory response. They exhibit functional plasticity, which is influenced by metabolic reprogramming, such as the induction of glycolysis in response to IFNγ, enhancing their cytokine secretion capabilities [93]. In allergic airway inflammation, macrophages and DCs show model-specific accumulation and phenotypic alterations, with increased expression of major histocompatibility complex class II and co-stimulatory molecules like CD80 and CD86, particularly in HDM-mediated models [94]. In COPD, monocyte-derived alveolar macrophages are key drivers of inflammation and tissue remodeling, which is characterized by a pro-inflammatory gene signature and significant recruitment in response to smoking [95]. Furthermore, macrophages contribute to immune cell recruitment, cytokine release, and airway remodeling in asthma, highlighting their role in both the exacerbation and resolution of inflammation [96]. The detection of macrophage subpopulations using radiolabeled techniques has shown promise in enhancing the sensitivity of imaging inflammatory processes for chronic respiratory diseases, indicating their potential as targets for therapeutic interventions [97]. Collectively, the results of these studies underscore the complex interplay between macrophages and other immune cells in respiratory inflammation, emphasizing the importance of the metabolic and functional plasticity of macrophages in disease pathogenesis and potential therapeutic targeting. Recent scRNA-seq analysis of Th cells isolated from the airways, lung tissue, and lymph nodes of an HDM-induced allergic asthma mouse model revealed a significant presence of Treg, Th1, Th2, and Th17 cell subpopulations, as well as upregulated expression of genes associated with Th2 cell differentiation and activation, including *CD200R1*, *IL6*, *PLAC8*, and *IGFBP7*, in the airways. Additionally, the results of the analysis demonstrated that carbohydrate and lipid metabolism play a crucial role in promoting Th2-mediated immune responses [98]. Seumois et al. conducted a single-cell transcriptomic analysis of diverse HDM-reactive Th cells and Treg cells, and found that *TNFSF10* expression suppresses Th cell activation, highlighting its potential role in inhibiting allergic responses within specific T lymphocyte subsets [99]. Aranda et al. investigated the relationship between memory B cells and IgE production by performing a scRNA-seq analysis of peripheral blood B lymphocytes obtained from patients with allergic conditions such as asthma. Their study identified an increase in a CD23⁺IL4R⁺IgG⁺ memory B cell subset, regulated by IL4/IL13 gene expression, which was found to be associated with IgE production. These findings suggest that this particular memory B cell population plays a key role in IgE production [100].

## Signaling pathways involved in asthma

Cellular signaling networks constitute the molecular basis for the pathophysiological changes observed in asthma. These intricate pathways translate external stimuli into specific cellular responses that drive the development of the hallmark features of asthma. Recent advances in molecular biology and systems pharmacology have significantly enhanced our understanding of how these interconnected signaling cascades coordinate the complex interplay between immune cells, structural cells, and the airway microenvironment.

Mechanistic investigation of these pathways has revealed targetable nodes for therapeutic intervention beyond conventional treatments. By dissecting the molecular switches that control airway inflammatory responses, oxidative damage, and structural remodeling, researchers are identifying novel therapeutic opportunities that may address the unmet needs in the management of severe and therapy-resistant asthma. The major signaling networks implicated in the pathogenesis of asthma are systematically discussed in the subsequent sections, with particular focus on oxidative stress and inflammatory pathways that represent promising targets for next-generation asthma therapeutics.

### Signaling pathways involved in oxidative stress in asthma

Oxidative stress plays a significant role in the pathophysiology of asthma, influencing various signaling pathways that contribute to inflammation and airway remodeling. The key signaling pathways associated with oxidative stress in asthma include the Nrf2, NFκB, mitochondrial, MAPK, thioredoxin-1, and aldose reductase pathways. These pathways are crucial for mediating the oxidative stress response and inflammation in asthmatic conditions. Table 1 provides a comprehensive summary of these pathways, including their key components, regulatory mechanisms, and therapeutic implications. Details of these pathways and their significance in asthma pathogenesis and potential therapeutic strategies are described in the following sections.

**Table 1:** Key signaling pathways involved in oxidative stress in asthma: molecular components and therapeutic implications

Signaling pathway	Key components	Regulatory mechanisms	Therapeutic implications	References
Nrf2 Pathway	• Nrf2 transcription factor• Keap1 inhibitor • Antioxidant response elements (AREs) • Heme oxygenase 1 (HO-1) • Glutathione peroxidase	• Keap1 inhibits Nrf2 under normal conditions • Oxidative stress disrupts Keap1-Nrf2 binding • Nrf2 translocates to nucleus and activates AREs • Enhances expression of antioxidant enzymes	• Dexmedetomidine activates Nrf2 pathway • Tectorigenin activates Keap1/Nrf2/HO-1 signaling • Neutrophil elastase inhibitors activate Keap1/Nrf2 • Nrf2 activation suppresses allergic lung inflammation	[101–107]
NFκB Pathway	• NFκB transcription factor • IκBα inhibitor • Malondialdehyde (MDA) • Glutathione	• Redox-sensitive transcription factor • Oxidative stress induces NFκB activation • Degradation of IκBα leads to NFκB nuclear translocation • Regulates pro-inflammatory cytokine expression	• N-acetylcysteine reduces NFκB activation • Dexmedetomidine modulates NFκB pathway • Positive correlation between MDA levels and NFκB activation • Obesity exacerbates NFκB activation	[108–111]
Mitochondrial Pathway	• Reactive oxygen species (ROS) • ATP synthase • NLRP3 inflammasome • ULK1/Atg9a/Rab9 pathway • Calcium homeostasis	• Mitochondrial dysfunction increases ROS production • ROS triggers inflammasome activation • ULK1 activation promotes inflammasome activation • Golgi apparatus fragmentation intensifies inflammation	• MitoQuinone reduces protein oxidation • SS-31 alleviates airway inflammation • Mitochondrial-targeted antioxidants modulate function • Reduction of endoplasmic reticulum stress	[112–117]
MAPK Pathway	• ERK • JNK • p38 MAPK • Transcription factors	• Activated by cytokines, chemokines, and oxidative stress • Oxidative stress induces MAPK phosphorylation • Promotes expression of pro-inflammatory genes • H₂O₂ activates ERK1/2 and p38 MAPK	• Green tea extract inhibits MAPK phosphorylation • MAPK modulation reduces oxidative stress • Potential target in corticosteroid-insensitive asthma • Role in gene expression of surfactant proteins	[118–125]
Thioredoxin-1 Pathway	• Thioredoxin-1 protein • Redox homeostasis • Macrophage migration inhibitory factor (MIF)	• Scavenges reactive oxygen species • Inhibits MIF production • Reduces eosinophil recruitment • Decreases mucus metaplasia	• Acts locally without suppressing systemic immunity • Distinct from glucocorticoid mechanisms • Potential synergy with Keap1/Nrf2/HO-1 pathway • Addresses oxidative stress component of asthma	[105, 125–129]
Aldose Reductase (AR) Pathway	• Aldose reductase enzyme • Polyol pathway • NFκB and AP-1 transcription factors • Th2 cytokines	• Mediates oxidative stress-induced signaling • Regulates NFκB-dependent inflammatory markers • Amplifies inflammatory response in airways • Influences IgE production	• AR inhibitors suppress Th2 cytokines • Reduces airway hyperresponsiveness • Attenuates ROS production • Potential alternative in corticosteroid-insensitive cases	[132–137]

#### Nrf2 signaling pathway

The Nrf2 signaling pathway regulates oxidative stress in asthma by modulating the expression of cytoprotective genes. Nrf2, a transcription factor, is typically inhibited by Keap1 under normal conditions; however, oxidative stress disrupts this inhibition, allowing Nrf2 to translocate to the nucleus and activate elements of the antioxidant response that enhance the expression of antioxidant enzymes such as glutathione peroxidase and superoxide dismutase [101–103]. Oxidative stress is a significant contributor to inflammation and AHR in asthma, and activation of the Nrf2 pathway has been shown to alleviate these symptoms. For instance, a previous study demonstrated that dexmedetomidine, an α−2-adrenergic receptor agonist, reduced oxidative stress and airway inflammation in a murine asthma model by activating the Nrf2 pathway, which increases the expression of antioxidant genes such as heme oxygenase 1 (HO-1) and glutathione peroxidase 4 [104]. In another study, tectorigenin, an isoflavonoid, activated the Keap1/Nrf2/HO-1 signaling pathway in Th2-mediated allergic asthma models, thereby reducing oxidative stress and inflammation [105]. Moreover, neutrophil elastase inhibitors have been shown to activate the Keap1/Nrf2 pathway in obese asthmatic rats, thereby enhancing antioxidant enzyme activity and reducing oxidative stress [106]. The therapeutic potential of Nrf2 activators in asthma is further supported by the results of studies indicating that Nrf2 activation can suppress allergic lung inflammation by modulating the type 2 innate lymphoid cell response, which is crucial in the allergic immune response [107].

#### NFκB signaling pathway

The NFκB signaling pathway plays a crucial role in regulating oxidative stress in asthma because it is a redox-sensitive transcription factor that modulates inflammatory gene expression in response to oxidative stress. Oxidative stress contributes to airway inflammation in asthma by inducing the activation of NFκB, which in turn regulates the expression of pro-inflammatory cytokines and other mediators involved in airway pathology [108, 109]. Activation of NFκB is strongly associated with oxidative stress markers such as malondialdehyde (MDA) and glutathione (GSH). Studies have shown that MDA level is positively correlated with NFκB activation in the lung tissues of asthmatic models. In particular, increased MDA and decreased GSH levels, alongside elevated NFκB activity, were observed in studies of obesity–asthma models, demonstrating that obesity exacerbates oxidative stress and NFκB activation. The NFκB pathway is activated through the degradation of its inhibitor, IκBα, and subsequent translocation of NFκB to the nucleus, where it promotes the transcription of inflammatory genes [110, 111]. Therapeutic strategies that target NFκB, such as the use of N-acetylcysteine, have shown promise in reducing oxidative stress and NFκB activation more effectively than traditional treatments such as budesonide [111]. The interplay between NFκB and other signaling pathways, such as the Nrf2 pathway, highlights the complex regulatory mechanisms involved in oxidative stress and inflammation in asthma. Notably, dexmedetomidine has been shown to exert antioxidative effects by modulating both the NFκB and Nrf2 pathways [104]. This finding suggests the potential therapeutic benefits in dexmedetomidine in the management of asthma symptoms.

#### Mitochondrial signaling pathway

In asthma, oxidative stress is intricately regulated by mitochondrial signaling pathways, which play crucial roles in the pathophysiology of the disease. Mitochondria are central to the production of ROS, which triggers inflammatory pathways and contributes to oxidative stress in the airways. This is particularly evident in asthma, in which mitochondrial dysfunction leads to increased ROS production, reduced ATP synthase activity, and abnormal calcium homeostasis, all of which exacerbate airway inflammation and remodeling [112, 113]. The mitochondrial signaling pathways involved in asthma include the activation of inflammasomes, such as the NLRP3 inflammasome, which is triggered by mitochondrial ROS and contributes to the inflammatory cascade in the bronchial epithelium [114, 115]. Additionally, the ULK1/Atg9a/Rab9 signaling pathway has been identified as a key regulator of mitochondrial oxidative stress and inflammation in asthma, with ULK1 activation promoting inflammasome activation and Golgi apparatus fragmentation, further intensifying pulmonary inflammation [115]. Therapeutic interventions that target mitochondrial oxidative stress, such as the use of mitochondrial-targeted antioxidants like MitoQuinone (MitoQ) and SS-31, have shown promise in reducing airway inflammation and oxidative stress by modulating mitochondrial function and signaling pathways. For instance, MitoQ has been shown to effectively attenuate airway hyperreactivity and remodeling in both lean and obese models of allergic asthma by reducing protein oxidation and endoplasmic reticulum stress [116]. Similarly, SS-31 has been shown to alleviate cigarette smoke-induced airway inflammation and oxidative stress by modulating mitochondrial dynamics and inhibiting the MAPK signaling pathway [117].

#### Mitogen-activated protein kinase signaling pathway

The mitogen-activated protein kinase (MAPK) signaling pathway plays a vital role in the modulation of oxidative stress in asthma, influencing both inflammatory and structural changes in the airways. Oxidative stress, characterized by an imbalance between ROS and antioxidant defenses, is a significant pathogenic factor in asthma that leads to the activation of MAPK pathways, including the ERK, JNK, and p38 MAPK pathways, which are pivotal in mediating inflammatory responses [118–120]. These pathways are activated by various extracellular stimuli, such as cytokines, chemokines, and oxidative stress, which are prevalent in asthmatic conditions [119, 121]. Specifically, oxidative stress-induced activation of MAPK pathways can lead to phosphorylation of transcription factors and other signaling molecules, thereby promoting the expression of pro-inflammatory genes and contributing to airway inflammation and remodeling [121, 122]. For instance, hydrogen peroxide, a common ROS, activates ERK1/2 and p38 MAPK, which are involved in the regulation of gene expression related to surfactant proteins in lung epithelial cells. This function highlights the role of MAPK in oxidative stress responses [123]. Furthermore, the therapeutic potential of targeting MAPK pathways is underscored by the findings from research that showed interventions such as green tea extract can suppress airway inflammation by inhibiting MAPK phosphorylation, thereby reducing oxidative stress and its downstream effects [124]. This suggests that modulating MAPK signaling could be a promising strategy for managing oxidative stress and inflammation in asthma, especially in cases where traditional therapies are less effective due to corticosteroid insensitivity, which is linked to oxidative stress [122, 125].

#### Thioredoxin-1 signaling pathway

The Thioredoxin-1 (Trx1) signaling pathway modulates oxidative stress in asthma, primarily through its antioxidative and anti-inflammatory properties. Trx1 is a redox-active protein that helps maintain cellular redox homeostasis and is upregulated in response to oxidative stress, which is a significant factor in the pathogenesis of asthma [126, 127]. Trx1 exerts its protective effects by scavenging ROS, thereby reducing oxidative damage and inflammation in the respiratory system [127]. It also inhibits the production of macrophage migration inhibitory factor (MIF), a key modulator of airway inflammation, which leads to decreased recruitment of eosinophils and reduced mucus metaplasia in the lungs [126, 128]. This mechanism is distinct from those of traditional anti-inflammatory agents such as glucocorticoids because Trx1 does not suppress systemic Th1/Th2 immune responses but rather acts locally to mitigate inflammation [128]. Trx1 shares a similar redox-active motif to that of MIF and can directly bind to both intracellular and extracellular MIF, thereby suppressing pro-inflammatory signaling pathways such as ERK1/2 and p38 MAPK. Trx1 is capable of suppressing the pro-inflammatory activation of immune cells, including eosinophils and monocytes, through this mechanism in the respiratory system [126]. The importance of oxidative stress in asthma is further highlighted by evidence from studies that proved oxidative stress can alter immune responses, such as the Th1/Th2 balance, and activate pro-inflammatory pathways such as NFκB, which exacerbate airway inflammation [125, 129]. A recent study showed that Trx1 attenuates cigarette smoke extract-induced endoplasmic reticulum (ER) stress, ROS production, mitochondrial dysfunction, and mitophagy-mediated inflammasome activation while enhancing the activation of the Nrf2/HO-1 signaling pathway, contributing to the improved antioxidant and anti-inflammatory response in murine lungs [130]. Similarly, Trx1 suppressed NLRP3 inflammasome activation and pro-IL1β production by inhibiting the IRE1α signaling pathway-mediated ER stress, thereby attenuating cellular ROS generation and mitigating the inflammatory response [131]. Additionally, the Keap1/Nrf2/HO-1 signaling pathway, which is activated by antioxidants such as tectorigenin, plays a role in reducing oxidative stress and inflammation in asthma, suggesting a potential therapeutic synergy with Trx1 [105].

#### Aldose reductase signaling pathway

The aldose reductase (AR) signaling pathway plays a significant role in modulating oxidative stress in asthma through its involvement in inflammatory processes. AR, an enzyme initially recognized for its role in the polyol pathway, has been identified as a key mediator of oxidative stress-induced molecular signaling, which is crucial in the pathogenesis of asthma [132, 133]. In asthma, oxidative stress is a major contributor to airway inflammation, often exacerbated by ROS that activates redox-sensitive transcription factors such as NFκB and AP-1, leading to the expression of pro-inflammatory cytokines and chemokines [134, 135]. AR influences these pathways by regulating the NFκB-dependent expression of inflammatory markers, thereby amplifying inflammatory response in the airways [132, 134]. Inhibition of AR has been shown to suppress the expression of Th2 cytokines and reduce airway hyperresponsiveness, IgE levels, and eosinophil infiltration in animal models of asthma, which suggests its potential as a therapeutic target [136]. Furthermore, AR inhibitors have demonstrated efficacy in preventing oxidative stress-induced activation of NFκB and AP-1, which are critical in the inflammatory cascade associated with asthma [135]. The therapeutic potential of AR inhibitors is further supported by their ability to attenuate ROS production, thereby disrupting the signaling cascades that lead to inflammation [137]. Given that clinical trials on the role of AR inhibitors in diabetic complications have proven that AR inhibitors have a manageable side effect profile, they present a promising avenue for developing new treatments for asthma [132]. Moreover, this approach could complement existing therapies, particularly in cases where corticosteroid insensitivity is an issue due to oxidative stress [81].

### Signaling pathways involved in inflammation in asthma

The pathophysiology of asthma is closely linked to various signaling pathways that mediate inflammation. Evaluation of these pathways is crucial for understanding the mechanisms of asthma and developing targeted therapies. The characteristic inflammation in asthma is primarily driven by immune responses, particularly those that involve Th cells and various cytokines. These responses are mediated through several key signaling pathways, including JAK-STAT, NFκB, inflammasome, toll-like receptors (TLRs)/NLRs, MAPK, and GPCR pathways, each contributing to the progression and severity of the disease. Table 2 provides a comprehensive summary of these inflammatory signaling pathways, their key molecular components, regulatory mechanisms, and potential therapeutic implications. Outlined below are detailed descriptions of the main signaling pathways associated with inflammation in asthma.

**Table 2:** Key signaling pathways involved in inflammation in asthma: molecular components and therapeutic implications

Signaling pathway	Key components	Regulatory mechanisms	Therapeutic implications	References
JAK-STAT Pathway	• JAK kinases • STAT transcription factors • IL4, IL5, IL13 cytokines • Th2 cells	• Cytokine binding activates JAK phosphorylation • JAK phosphorylates STATs, causing dimerization • STAT dimers translocate to nucleus • Induces transcription of genes promoting inflammation	• Inhaled pan-JAK inhibitor LAS194046 reduces inflammation • Selective JAK1 inhibitors (AZD0449, AZD4604) • Inhibition of STAT phosphorylation in lung tissues • Particularly effective in high Th2 activity asthma	[138–142]
NFκB Pathway	• NFκB transcription factor • IκBα inhibitory protein • GM-CSF • IL8	• Inflammatory signals activate NFκB • IκBα degradation allows NFκB nuclear translocation • Induces pro-inflammatory cytokine expression • Modulates airway smooth muscle cell phenotypes	• Persistent activation in severe asthma despite glucocorticoids • Contributes to airway remodeling and hyperresponsiveness • Interacts with endoplasmic reticulum stress pathway • Impacts viral infection responses and epithelial barrier function	[143–147]
Inflammasome Pathway	• NLRP3 inflammasome • Caspase-1 • IL1β, IL18 cytokines • Toll-like receptors (TLRs)	• Environmental allergens trigger NLRP3 activation • Caspase-1 cleaves pro-IL1β into active form • Promotes Th17 cell differentiation • Enhances neutrophil recruitment	• Critical in severe steroid-resistant asthma (SSRA) • Linked to glucocorticoid resistance • Influences macrophage polarization (M2 phenotype) • Facilitated by cell-in-cell structures for intercellular communication	[148–155]
TLRs and NLRs Pathway	• TLRs • NOD-like receptors (NLRs) • PI3K/Akt pathway • JNK pathway • TRIF-dependent pathway	• TLRs recognize allergens and microbial products • TLR2 regulates inflammation via PI3K/Akt pathway • TLR9 suppresses melatonin biosynthesis via JNK • TRIF-dependent pathway of TLR4 modulates CD4^+^ T cells	• TLR2 deficiency mitigates inflammation • TLR9 deficiency restores melatonin levels • TLR7 activation shifts from Th2 to Th1 response • Dual role in either exacerbating or mitigating inflammation	[153, 156–162]
MAPK Pathway	• p38 MAPK • ERK • JNK • MAP3K19	• Regulates cell growth, survival, differentiation, apoptosis • MAP3K19 modulates epithelial response to TWEAK and TGFβ1 • TLR2 activates MAPK signaling • Influences pro-inflammatory cytokine production	• Sakuranetin reduces inflammation by inhibiting ERK1/2, JNK, p38 • Apigenin alleviates inflammation and epithelial cell apoptosis • Reduces phosphorylation of MAPKs • Modulates apoptosis-related proteins	[119, 153, 163–166]
GPCR Pathway	• G-protein-coupled receptors • G-protein-coupled estrogen receptor (GPER) • Prostaglandin D2 receptor 2 (DP2) • GPR120 receptor • β2-adrenergic receptors • Regulator of G protein signaling 2 (RGS2)	• GPER activation suppresses airway inflammation • DP2 mediates type 2 immune cell recruitment • GPR120 stimulation attenuates airway remodeling • β2-adrenergic receptors regulate bronchodilation • RGS2 reduces Gq-coupled receptor activity	• GPER agonist G-1 reduces inflammatory cell accumulation • DP2 antagonists reduce eosinophilic inflammation • GPR120 inhibits STAT6 and Akt pathways • β2-adrenergic receptors are common therapeutic targets • Multiple GPCR targets to modulate inflammation	[167–173]

#### JAK-STAT signaling pathway

The JAK-STAT signaling pathway regulates inflammation in asthma by modulating various cytokine-mediated immune responses. This pathway is integral to the pathogenesis of asthma, particularly in the differentiation and function of Th2 cells, which are central to the inflammatory processes in asthma. The JAK-STAT pathway is activated by cytokines such as IL4, IL5, and IL13, which are pivotal in allergic asthma, leading to the transcription of genes that promote inflammation and AHR [138, 139]. Inhibition of this pathway has shown some potential in reducing airway inflammation and improving lung function. For instance, a previous study indicated that inhaled pan-JAK inhibitor LAS194046 reduced allergen-induced airway inflammation and late asthmatic response in a rat model, highlighting the potential of localized JAK inhibition to mitigate lung inflammation without significant systemic exposure [140]. Inhaled forms of selective JAK1 inhibitors, such as AZD0449 and AZD4604 have been developed, and they have shown efficacy in reducing lung inflammation and late asthmatic response by inhibiting STAT phosphorylation in lung tissues [141]. Furthermore, the role of the JAK-STAT pathway extends to the regulation of mast cells, which are key mediators of allergic responses in asthma. The involvement of STAT5 in mast cell proliferation and mediator release highlights the importance of this pathway in maintaining immune homeostasis [142]. Therefore, therapeutic targeting of the JAK-STAT pathway to attenuate the inflammatory signaling cascades that exacerbate asthma represents a promising strategy for managing the disease, especially in severe cases characterized by high Th2 activity.

#### NFκB signaling pathway

The NFκB signaling pathway regulates inflammation in asthma by modulating the expression of various inflammatory cytokines and mediators. In asthma, NFκB is activated in response to inflammatory signals, leading to the degradation of its inhibitory protein, IκBα, and subsequent translocation of NFκB to the nucleus, where it induces the expression of pro-inflammatory cytokines such as granulocyte–macrophage colony-stimulating factor and IL8, which are overexpressed in asthma. This persistent activation of NFκB is particularly evident in severe asthma, where it perpetuates the production of inflammatory mediators despite the patient receiving glucocorticoid treatment [143]. Additionally, NFκB is involved in the modulation of airway smooth muscle cell phenotypes, contributing to airway remodeling and hyperresponsiveness, which are hallmarks of asthma [144]. The pathway is also implicated in the exacerbation of asthma symptoms through its interaction with other signaling pathways, such as the endoplasmic reticulum stress pathway, which can be triggered by environmental factors such as diisononyl phthalate exposure [145]. Furthermore, NFκB signaling plays an important role in response to viral infections, such as those caused by rhinoviruses, which can exacerbate asthma by impairing antiviral responses and enhancing Th2 cytokine expression [146]. In allergic asthma, activation of NFκB in airway epithelial cells leads to epithelial plasticity and barrier disruption, facilitating allergen penetration and sensitization [147].

#### Inflammasome signaling pathway

The inflammasome signaling pathway regulates inflammation in asthma, particularly through activation of the NLRP3 inflammasome. This pathway contributes to both the development and exacerbation of asthma by mediating the release of pro-inflammatory cytokines such as IL1β and IL18, which are critical in the pathogenesis of severe steroid-resistant asthma (SSRA) and other asthma phenotypes [148, 149]. Activation of the NLRP3 inflammasome is often triggered by environmental allergens, leading to the cleavage of pro-IL1β into its active form via caspase-1, which subsequently promotes a pro-inflammatory milieu conducive to Th17 cell differentiation and neutrophil recruitment [150, 151]. This process is further exacerbated by the involvement of TLRs, particularly TLR3 and TLR4, which enhance activation of the inflammasome and the subsequent inflammatory response through pathways such as the NFκB and MAPK pathways [152, 153]. The role of the NLRP3 inflammasome in asthma is not limited to cytokine production; it also influences macrophage polarization, particularly promoting the M2 phenotype through IL4 upregulation, which contributes to the chronic inflammation observed in asthma [154]. Moreover, activation of the inflammasome is linked to glucocorticoid resistance in SSRA, highlighting its potential as a therapeutic target to improve the outcomes of severe asthma [148]. Involvement of the inflammasome in asthma is further complicated by cell-in-cell structures that facilitate intercellular communication and exacerbate inflammation through the transfer of pro-inflammatory substances [155].

#### Toll-like receptors and NOD-like receptors signaling pathway

TLRs and NOD-like receptors (NLRs) play significant roles in asthma by modulating immune responses and inflammation through various signaling pathways. TLRs, which are innate pattern recognition receptors, recognize allergens and microbial products, thereby influencing allergic sensitization and airway inflammation. For instance, TLR2 regulates allergic airway inflammation through the PI3K/Akt signaling pathway, which is associated with autophagy in asthma models. Activation of this pathway leads to increased inflammatory cell infiltration and cytokine production, which are mitigated in TLR2-deficient mice. This highlights the importance of TLR2 in asthma pathogenesis [156]. Similarly, TLR9 contributes to allergic airway inflammation by suppressing melatonin biosynthesis through the JNK pathway. Conversely, TLR9 deficiency reduces inflammation and restores melatonin levels [157]. TLR4, through its TRIF-dependent pathway, can protect against allergic airway disease by modulating CD4^+^ T cell responses, a function that represents a potential therapeutic target for asthma management [158]. Additionally, the interaction between TLR4 and sphingosine-1-phosphate (S1P) exacerbates allergic responses, indicating its role in enhancing airway inflammation [159]. The NFκB and MAPK pathways are also implicated in TLR2-mediated inflammation. Findings from previous studies indicated reduced levels of inflammatory markers in TLR2 knockout mice, suggesting the involvement of these pathways in asthma [153]. Furthermore, TLR7 activation can shift the inflammatory response from a Th2 to a Th1 pattern, offering a potential immunomodulatory approach in asthma treatment [160]. The complex interplay between TLRs and their signaling pathways underscores their dual roles in asthma, either exacerbating or mitigating inflammation depending on the context and the specific receptor involved [161, 162].

#### MAPK signaling pathway

Evidence from several studies has demonstrated that the MAPK signaling pathway regulates inflammation in asthma. MAPKs, including p38, ERK, and JNK, are integral to the cellular processes that mediate inflammatory responses in asthma, influencing cell growth, survival, differentiation, and apoptosis [163]. A previous study showed that MAP3K19, a component of the MAPK pathway, suppressed allergic airway inflammation in asthma models by modulating epithelial response to stimuli such as TWEAK and TGFβ1, thereby reducing the production of pro-inflammatory cytokines such as RANTES (CCL5) [164]. The pathway's involvement is further highlighted by the role of TLR2, which regulates allergic airway inflammation through both the NFκB and MAPK signaling pathways, suggesting that inhibiting these pathways can alleviate inflammation in asthmatic mice [153]. Additionally, therapeutic interventions that target MAPK signaling, such as the use of sakuranetin, have demonstrated efficacy in reducing eosinophilic lung inflammation and Th2/Th17 cytokine production by inhibiting the activation of ERK1/2, JNK, and p38 MAPKs [165]. Apigenin alleviates airway inflammation and epithelial cell apoptosis in allergic asthma by inhibiting the MAPK pathway, reducing the phosphorylation of ERKs, JNKs, and p38 MAPKs, and modulating apoptosis-related proteins [166]. These findings underscore the potential of targeting the MAPK signaling pathway as a therapeutic strategy for managing asthma, given its central role in mediating inflammatory and structural changes in the airways [119].

#### G-protein-coupled receptor signaling pathway

The regulation of inflammation in asthma through G-protein-coupled receptors (GPCRs) involves several pathways, each contributing to the modulation of immune responses and airway remodeling. The G-protein-coupled estrogen receptor (GPER) has been shown to play a significant role in suppressing airway inflammation in asthma models. Activation of GPER by the agonist G-1 reduces inflammatory cell accumulation and Th2 cytokine levels, while increasing anti-inflammatory cytokines such as IL10, which highlights its potential as a therapeutic target for chronic allergic asthma [167, 168]. Similarly, the prostaglandin D2 (PGD2) receptor 2 (DP2) pathway is crucial in asthma pathophysiology as it mediates the recruitment and activation of type 2 immune cells, leading to inflammation and tissue remodeling. Antagonists that target the DP2 receptor have shown promise in reducing eosinophilic inflammation and improving asthma symptoms; however, the clinical efficacy of these agents varies across patient cohorts [169, 170]. Additionally, stimulation of the GPR120 receptor can attenuate airway remodeling and inflammation by inhibiting pathways such as the STAT6 and Akt pathways, which are involved in cytokine-induced epithelial injury [171]. The role of GPCRs extends to the regulation of bronchoconstriction and bronchodilation, with β2-adrenergic receptors typically targeted by common asthma therapeutics to enhance bronchodilation and reduce airway resistance [172]. Furthermore, the regulator of G protein signaling 2 is implicated in the modulation of GPCR signaling, particularly the reduction of Gq-coupled receptor activity, which is associated with bronchoconstriction and inflammation in asthma [173].

## Naturally derived bioactive compounds as potential regulators of oxidative stress and inflammation

The limitations of conventional asthma therapies, such as corticosteroid resistance and adverse effects with long-term use, have prompted research into alternative therapeutic approaches. Natural bioactive compounds have emerged as promising candidates for asthma management owing to their multifaceted effects on inflammation and oxidative stress pathways. These compounds, which are derived from various plants, herbs, and food sources, offer a mechanistic advantage through their ability to modulate multiple molecular targets simultaneously. Recent advances in phytochemistry and molecular pharmacology have enabled researchers to identify specific compounds that effectively target the key signaling pathways involved in the pathogenesis of asthma. These natural agents have demonstrated a remarkable ability to suppress pro-inflammatory mediators, balance Th1/Th2 responses, scavenge ROS, and enhance antioxidant defense mechanisms in experimental models of asthma. Table 3 presents a comprehensive summary of these natural compounds, their sources, key mechanisms of action, and supporting experimental evidence. The most promising natural compounds with proven efficacy in modulating asthma-related inflammatory and oxidative stress pathways are discussed in the subsequent sections.

**Table 3:** Natural bioactive compounds for asthma management: sources, mechanisms, and evidence

Compound	Source	Key mechanisms	Experimental evidence	References
d-α-tocopheryl acetate	Natural-source vitamin E	• Antioxidant properties • Decreases oxidant stress markers (F2-isoprostanes) • Reduces inflammatory cytokines (IL3, IL4) • Increases IL12 levels • Inhibits 5-lipoxygenase pathway	• Improved airway responsiveness to methacholine in atopic asthmatics • Inhibited leukotriene synthesis in a concentration-dependent manner	[174–178]
Phycocyanobilin	Spirulina	• Mimics biliverdin/bilirubin effects on NADPH oxidase • Enhances antioxidant defense system • Reduces airway inflammation • Increases IL12 production	• Prevented oxidative stress and inflammation in ovalbumin (OVA)-induced rat asthma model • Suppressed airway hyperresponsiveness in allergic asthma models	[179–184]
2,3,5,4′-tetrahydroxystilbene-2-O-β-d-glucoside (TSG)	Polygonum multiflorum	• Suppresses Th2 cytokines (IL4, IL5) • Enhances Th1 responses (IFNγ) • Reduces total IgE and OVA-specific IgE • Modulates TGFβ1 signaling • Inhibits arginase activity	• Rebalanced Th1/Th2 immune response in OVA-induced asthma mouse model • Reduced airway inflammation and remodeling	[185–188]
Quercetin	Flavonoid from fruits and vegetables	• Inhibits ferroptosis via SIRT1/Nrf2/HO-1 pathway • Downregulates TGFβ1/Smad pathway • Reduces periostin levels • Regulates Th1/Th2 balance • Inhibits histamine production	• Reduced airway inflammation, fibrosis, and hyperreactivity in asthmatic mice • Clinical studies with Quercefit™ showed reduced symptoms and oxidative stress	[189–193]
Resveratrol	Polyphenol from grapes, berries	• Inhibits cellular infiltration and mucus production • Relaxes smooth muscles • Prevents fibrosis • Modulates gut-lung axis • Inhibits HMGB1/TLR4/NFκB pathway • Activates Keap-1/Nrf2 defense system	• Improved pulmonary functions • Decreased lung inflammation • Increased beneficial gut bacteria • Enhanced barrier functions in lungs • Reduced airway remodeling	[194–198]
Curcumin	Turmeric (Curcuma longa)	• Suppresses NFκB, STAT6, GATA3, and Wnt/β-catenin signaling • Downregulates Th2 cytokines (IL4, IL5, IL13) • Inhibits epithelial-mesenchymal transition • Attenuates airway hyperresponsiveness	• Reduced airway inflammation and remodeling • Improved bioavailability with novel delivery systems • Reversed mucus hypersecretion	[199–210]
Baicalin	Scutellaria baicalensis	• Downregulates Th2 cytokines and transcription factors • Modulates TLR4/NFκB axis • Inhibits airway smooth muscle cell proliferation • Targets multiple asthma-related genes	• Suppressed eosinophilic infiltration and mucus hypersecretion • Reduced proinflammatory signaling and oxidative stress • Prevented airway remodeling	[2, 211–214]
Formononetin	Isoflavone from legumes	• Reduces serum IgE levels and cytokines (IL4, IL6, IL17A) • Promotes epithelial barrier repair • Inhibits ESR1/NLRP3/Caspase-1 signaling • Regulates endoplasmic reticulum-stress transcription factor XBP-1 • Inhibits mast cell degranulation	• Reduced airway inflammation in house dust mite-induced asthmatic mice • Decreased IgE production in B cells • Improved lung function and reduced oxidative stress in murine models	[215–219]
Allicin	Garlic (Allium sativum)	• Anti-inflammatory actions • Antimicrobial properties • Reduces inflammatory cell infiltration • Modulates cytokine profiles	• Ameliorated allergic airway inflammation • Reduced mucus hypersecretion • Inhibited growth of lung pathogenic bacteria • Reduced leukocyte infiltration and improved lung structure	[220–223]
Tectorigenin	Methoxy-isoflavone from Belamcanda chinensis	• Reduces eosinophil infiltration and serum IL5 • Modulates Th1/Th2 balance • Activates Keap1/Nrf2/HO-1 pathway • Inhibits TGFβ1/Smad and TLR4/NFκB signaling	• Reduced inflammatory response in lungs • Enhanced antioxidant defenses • Prevented pulmonary fibrosis and airway inflammation	[105, 182, 224, 225, 225]
Salidroside	Rhodiola rosea	• Reduces pro-inflammatory cytokines (IL4, IL5, IL13) • Increases anti-inflammatory cytokines (IFNγ, IL10) • Regulates Th1/Th2 balance• Inhibits GATA3 and enhances T-bet mRNA expression • Inhibits NFκB activation	• Reduced eosinophil counts in bronchoalveolar lavage fluid • Effects similar to dexamethasone • Protective effects against acute lung injury • Mitigated pulmonary inflammation in chronic obstructive pulmonary disease models	[226–230]
Naringenin	Flavonoid from citrus fruits	• Antioxidant and anti-inflammatory properties • Suppresses IL5 and ROS production • Modulates NFAT and Nrf2 pathways • Protects airway cilia• Bronchodilatory effects	• Improved allergic asthma symptoms in rat models • Maintained mucociliary clearance • Relaxed airway smooth muscle cells • Decreased airway hyper-responsiveness and remodeling	[231–235]
Farnesol	Sesquiterpene alcohol from herbal plants	• Decreases IL6/IL10 level ratios • Restores cytokine secretion by macrophages • Increases IL10 levels from splenocytes • Modulates inflammatory mediators (COX2, TNFα)	• Reduced inflammation in OVA-sensitized asthmatic mice • Systemic antiallergic effects	[236, 237, 237]

### d-α-tocopheryl acetate

D-α-tocopheryl acetate, a natural-source form of vitamin E, has shown potential efficacy in modulating asthma symptoms, primarily through its antioxidant properties. In a study of patients with atopic asthma, supplementation with d-α-tocopheryl acetate for 16 weeks resulted in a significant decrease in the levels of oxidant stress markers, such as F2-isoprostanes, a reduction in allergen-induced inflammatory cytokines such as IL3 and IL4, and increased IL12 levels. These findings suggest that although d-α-tocopheryl acetate does not alter reactivity to specific allergens, it improves airway responsiveness to methacholine [174]. The role of tocopherol in asthma is further supported by its ability to inhibit the 5-lipoxygenase pathway, which is involved in leukotriene production, a key factor in asthma pathogenesis. In addition, the ability of tocopherol to inhibit leukotriene synthesis in a concentration-dependent manner highlights its potential as a therapeutic agent in asthma management [175]. However, the effects of vitamin E on asthma are complex, as different isoforms of vitamin E, such as α-tocopherol and γ-tocopherol, have opposing effects on lung inflammation. α-tocopherol appears to reduce lung inflammation, whereas γ-tocopherol may exacerbate it. Therefore, the isoform-specific effects of tocopherol must be considered in the treatment of asthma [176–178].

### Phycocyanobilin

Phycocyanobilin, a component derived from spirulina, has some potential in the management of asthma owing to its antioxidant and anti-inflammatory properties. The compound mimics the inhibitory effects of biliverdin/bilirubin on NADPH oxidase activity, which contributes to the pathogenesis of asthma by promoting airway smooth muscle hypercontractility and inflammation [179]. Studies on C-phycocyanin (CPC), a related compound, demonstrate its ability to prevent oxidative stress and inflammation in an ovalbumin-induced rat asthma model, suggesting that CPC can enhance the antioxidant defense system and reduce airway inflammation, thereby preventing asthma-induced airway remodeling [180]. Furthermore, R-phycocyanin has been shown to modulate immune responses by decreasing endocytosis and increasing IL12 production, which helped suppress AHR in allergic asthma models [181]. These findings are supported by results of research on the efficacy of phycocyanin in ameliorating colitis through phycocyanobilin-dependent antioxidant and anti-inflammatory mechanisms, indicating a similar potential for the treatment of respiratory conditions, including asthma [182]. Although direct clinical application of phycocyanobillin for asthma therapy requires further investigation, the existing preclinical evidence suggests that it could serve as a beneficial adjunct to conventional asthma therapies by targeting oxidative stress and inflammation pathways. This aligns with the broader trend of exploring natural bioactive compounds for asthma management, as with other phytoconstituents such as Pycnogenol and grape seed proanthocyanidin extract, which also exhibit anti-inflammatory and antioxidant effects [183, 184].

### 2,3,5,4′-tetrahydroxystilbene-2-O-β-d-glucoside

2,3,5,4′-tetrahydroxystilbene-2-O-β-d-glucoside (TSG), a primary compound derived from *Polygonum multiflorum*, exhibits promising therapeutic potential for asthma treatment. In an OVA-induced asthma mouse model, TSG significantly modulated immune responses by suppressing Th2 cytokines, such as IL4 and IL5, and reducing total IgE and OVA-specific IgE levels, which are typically elevated in asthma. Concurrently, TSG enhanced Th1 responses, indicated by increased IFNγ levels, suggesting rebalancing of the Th1/Th2 immune response, which is crucial in asthma management [185]. The anti-inflammatory and antioxidant properties of TSG, which have been highlighted in various studies, support its potential for the treatment of inflammatory diseases such as asthma [186]. Moreover, previous reports have indicated that TSG effectively mitigates pulmonary fibrosis through modulation of TGFβ1 signaling pathways and reduction of ROS, which may also contribute to its beneficial effects in asthma by reducing airway inflammation and remodeling [187]. In addition, TSG's ability to inhibit arginase activity and enhance nitric oxide production could improve vascular function, potentially alleviating asthma symptoms by improving blood flow in the airways and reducing oxidative stress [188].

### Quercetin

Quercetin, a naturally occurring flavonoid, has demonstrated significant potential in alleviating asthma symptoms through various mechanisms. It exhibits anti-inflammatory and antioxidative properties, which are crucial for managing asthma. In a study conducted using asthma models, quercetin inhibited ferroptosis via the SIRT1/Nrf2/HO-1 signaling pathway, thereby reducing chronic airway inflammation [189]. In another study, quercetin downregulated the TGFβ1/Smad pathway, which is associated with decreased periostin levels, leading to reduced airway inflammation, fibrosis, and hyperreactivity in asthmatic mice [190]. Quercetin has been observed to decrease the expression of pro-inflammatory cytokines, such as TNFα and IL1β, in rat models, while enhancing the expression of anti-inflammatory cytokines such as IL10, thus ameliorating oxidative stress and inflammation [191]. Furthermore, quercetin's ability to regulate Th1/Th2 balance and inhibit histamine production underscores its role in the management of allergic asthma [192]. Clinical studies conducted using quercetin formulated with the Phytosome® delivery system (Quercefit™) have shown that it can enhance standard asthma management by reducing symptoms, the need for rescue medication, and oxidative stress, while maintaining higher peak expiratory flow [193].

### Resveratrol

Resveratrol, which is a natural polyphenol, has demonstrated significant efficacy in alleviating asthma symptoms through various mechanisms. Resveratrol reduces inflammation by inhibiting cellular infiltration, oxidative stress, and mucus production, while relaxing smooth muscles and preventing fibrosis in the respiratory tract [194]. The effects of resveratrol on asthma are further enhanced by its ability to modulate the gut–lung axis, leading to improved pulmonary function and decreased lung inflammation. This is achieved by altering the microbiota, increasing beneficial bacteria such as *Akkermansia muciniphila*, and enhancing barrier functions in the lungs [195]. Additionally, resveratrol inhibits the HMGB1/TLR4/NFκB pathway, reducing airway inflammation and remodeling, which is crucial for managing asthma-induced airway changes [196]. Resveratrol has been observed to activate the Keap1/Nrf2 antioxidant defense system in obese–asthmatic models, thereby reducing oxidative stress and improving overall respiratory health [197]. Furthermore, resveratrol regulates miRNA-34a, which targets FoxP3, a key regulator of Treg cells, thus attenuating allergic asthma and the associated lung inflammation [198].

### Curcumin

Recent studies elucidated the therapeutic potential of curcumin, a pleiotropic phytochemical, in attenuating airway inflammation and remodeling associated with asthma [199–201]. Similarly, rationally designed delivery systems, including niosomes and synthetic curcuminoid analogues, have shown significantly improved bioavailability and target specificity, enhancing pharmacodynamic impact in asthma models [202–204]. Recent preclinical studies employing murine models of allergic asthma have demonstrated that native and structurally modified forms of curcumin exert robust anti-inflammatory and immunomodulatory effects [200, 202, 205, 206]. Mechanistically, curcumin has been shown to suppress major pro-inflammatory pathways, including NFκB, STAT6, GATA3, and Wnt/β-catenin signaling, thereby downregulating the expression of Th2-associated cytokines such as IL4, IL5, and IL13 [199, 200, 207, 208]. Additionally, curcumin exhibited multifaceted activity profiles, including inhibition of epithelial-mesenchymal transition, attenuation of airway hyperresponsiveness, and reversal of mucus hypersecretion [205, 209, 210].

### Baicalin

Baicalin, a flavonoid isolated from Scutellaria baicalensis, has emerged as a promising immunomodulatory agent in asthma therapy, owing to its multifaceted regulatory effects on airway inflammation and remodeling. A recent study has revealed that baicalin attenuates type 2 immune responses by downregulating Th2 cytokines and transcription factors, including GATA3 and STAT6, thereby suppressing eosinophilic infiltration and mucus hypersecretion in asthmatic airways [211]. In pediatric asthma models, baicalin was shown to modulate the TLR4/NFκB axis, reducing proinflammatory signaling cascades, oxidative stress, and SMC abnormal proliferation [212]. Furthermore, Hu et al. demonstrated that baicalin inhibits airway smooth muscle cell proliferation and airway remodeling-associated gene expression, including IL13, vascular endothelial growth factor, TGFβ1, matrix metalloproteinases (MMP) 9, and tissue inhibitor of metalloproteinase 1 via the MAPK and RAS signaling pathways, implicating a role in preventing airway remodeling [213]. A systems pharmacology approach has identified the capacity of baicalin to target 48 asthma-related genes such as IFNγ, IL4, IL5, IL10, IL13, and TNFα and signaling pathways, reinforcing its polypharmacological properties [2]. Moreover, in OVA-induced murine asthma models, baicalin administration significantly suppressed NFκB activation and downstream cytokine production, including CCR7, CCL19, CCL21, and IL6, leading to reduced lung tissue inflammation [214].

### Formononetin

Formononetin is a bioactive isoflavone that has demonstrated significant therapeutic potential in the treatment of asthma by targeting various inflammatory and immune pathways. In a study conducted using HDM-induced asthmatic mice, formononetin reduced airway inflammation by decreasing serum IgE levels and cytokines such as IL4, IL6, and IL17A, while promoting epithelial barrier repair through the inhibition of the ESR1/NLRP3/Caspase-1 signaling pathways [215]. Additionally, formononetin decreases IgE production in B cells by regulating the ER-stress transcription factor XBP-1, which indicates its potential in managing IgE-mediated allergic responses [216]. Findings from computational studies further support the role of formononetin in preventing mast cell activation and IgE production, highlighting its impact on pathways such as PI3K-Akt and Th17 cell differentiation [217]. Moreover, formononetin inhibits both IgE-dependent and IgE-independent mast cell degranulation, reducing the release of inflammatory mediators and attenuating NFκB signaling, which is crucial in allergic reactions [218]. In murine models of allergic asthma, formononetin improved lung function, reduced oxidative stress, and inhibited NFκB and JNK pathways, further underscoring its anti-inflammatory and antioxidant properties [219].

### Allicin

Allicin, a sulfur-containing compound found in garlic (*Allium sativum*), exhibits potential therapeutic effects on asthma owing to its anti-inflammatory and antimicrobial properties. Asthma is characterized by chronic inflammation and oxidative stress in the airways and may benefit from the biological properties of garlic compounds, which can modulate these pathogenic mechanisms [220]. Studies have demonstrated that garlic extracts, particularly the water fraction, can ameliorate allergic airway inflammation by reducing inflammatory cell infiltration and mucus hypersecretion, as well as modulating cytokine profiles to favor anti-inflammatory responses [221]. Allicin also exhibits significant antimicrobial activity as it effectively inhibits the growth of lung pathogenic bacteria, including multidrug-resistant strains, suggesting its potential use in treating bacterial lung infections associated with asthma [222]. Additionally, garlic has been found to exert anti-inflammatory effects through the reduction of leukocyte infiltration and improvement of lung structure in models of lung damage, which further supports its role in the management of respiratory conditions such as asthma [223].

#### Tectorigenin

Tectorigenin, a methoxy-isoflavone, has shown significant potential in alleviating symptoms of asthma through its anti-inflammatory and antioxidative properties. In studies conducted using ovalbumin-induced asthma models, tectorigenin reduced eosinophil infiltration and serum IL5 levels, indicating its ability to control inflammation and eosinophilia-related issues in asthma [182]. Additionally, tectorigenin modulates the Th1/Th2 balance by increasing Th1-related factors and decreasing Th2-related factors, thereby reducing the inflammatory response in the lungs. It also activates the Keap1/Nrf2/HO-1 signaling pathway, enhancing antioxidant defenses and reducing oxidative stress markers such as ROS [105]. Furthermore, tectorigenin inhibits pulmonary fibrosis and airway inflammation by modulating the TGFβ1/Smad and TLR4/NFκB signaling pathways, highlighting its potential role in preventing the structural lung changes associated with chronic asthma [224]. In a study on acute lung injury, tectorigenin reduced the numbers of inflammatory cells and improved antioxidant enzyme activity, further confirming its protective effects on lung tissue [225].

#### Salidroside

Salidroside, a natural compound derived from *Rhodiola rosea*, may potentially relieve asthma through various mechanisms. Studies have demonstrated that salidroside significantly reduces airway inflammation and oxidative stress in asthmatic models by decreasing the levels of pro-inflammatory cytokines such as IL4, IL5, and IL13, while increasing the levels of anti-inflammatory cytokines, such as IFNγ and IL10, in bronchoalveolar lavage fluid [226]. Additionally, salidroside regulates the Th1/Th2 balance, which is crucial for managing allergic asthma, by reducing eosinophil counts and modulating cytokine levels, similar to the effects of dexamethasone [227]. Furthermore, the ability of salidroside to inhibit GATA3 and enhance T-bet mRNA expression suggests its role in modulating immune responses, thereby ameliorating asthma symptoms [228]. Beyond the mitigation of asthma symptoms, salidroside also exhibits protective effects against acute lung injury by reducing inflammatory cytokines and inhibiting NFκB activation [229]. Furthermore, salidroside mitigates pulmonary inflammation in COPD by inhibiting M1 macrophage polarization through deactivation of the JNK/c-Jun pathway [230].

#### Naringenin

Naringenin, a flavonoid found in citrus fruits, has shown significant efficacy in alleviating asthma symptoms through various mechanisms. Studies conducted using rat models have shown that naringenin possesses strong antioxidant and anti-inflammatory properties, which are crucial for reducing oxidative stress and inflammation in lung tissues, thereby improving allergic asthma symptoms [231]. In a murine model, naringenin effectively suppressed the production of IL5 and ROS, which are key contributors to asthmatic inflammation, by modulating signaling pathways such as the NFAT and Nrf2 pathways [232]. Additionally, the ability of naringenin to protect airway cilia from structural and functional damage induced by cigarette smoke extract suggests its potential to maintain mucociliary clearance and reduce airway obstruction [233]. Furthermore, naringenin exerts bronchodilatory effects by relaxing airway smooth muscle cells, which is essential for reducing bronchial airway resistance in patients with asthma [234]. In chronic asthma models, naringenin treatment resulted in decreased AHR, inflammation, and remodeling, alongside reduced levels of serum IgE and Th2 cytokines, all of which indicate its role in delaying airway remodeling and asthma progression [235].

#### Farnesol

Farnesol, a sesquiterpene alcohol found in various herbal plants, has shown promising efficacy in improving asthma symptoms through its anti-inflammatory and antiallergic properties. In a study of OVA-sensitized and challenged asthmatic mice, farnesol supplementation decreased the IL6/IL10 level ratios in bronchoalveolar lavage fluid, indicating a reduction in inflammation. It also restored the cytokine secretion abilities of peritoneal macrophages and increased the levels of IL10 secreted by splenocytes, suggesting systemic antiallergic effects [236]. The potential of farnesol in asthma treatment is further supported by its ability to modulate inflammatory mediators such as COX2 and TNFα, which are crucial in asthma pathophysiology [237].

## Molecular targets for therapeutic intervention in asthma

Based on the understanding of oxidative stress and inflammatory signaling pathways in asthma, several molecular targets have emerged as promising candidates for novel asthma treatments. Table 4 summarizes major molecular targets and their therapeutic agents for asthma management. Briefly, therapeutic interventions for asthma primarily target oxidative stress pathways through Nrf2 activators and mitochondrial antioxidants, while addressing inflammatory cascades via JAK-STAT inhibitors, NFκB modulators, and inflammasome inhibitors. Emerging approaches include MAPK inhibitors, GPCR modulators, and epigenetic regulators. Dual-acting compounds such as phosphodiesterase (PDE) 3/4 inhibitors offer particularly promising results by simultaneously addressing multiple pathological mechanisms. While many candidates remain in preclinical development, several agents such as Fevipiprant (DP2 antagonist) and Ensifentrine (PDE3/4 inhibitor) have advanced to late-stage clinical trials, potentially offering new therapeutic options for severe and therapy-resistant asthma.

**Table 4:** Molecular targets for therapeutic intervention in asthma

Target category	Specific target	Mechanism of action	Therapeutic agents	Potential benefits	References
Oxidative Stress Pathways	Nrf2	Enhances antioxidant enzyme expression	Sulforaphane, Bardoxolone methyl	Reduced oxidative stress, decreased airway inflammation	[101–107]
			Tectorigenin, Quercetin, Resveratrol	Reduced eosinophil infiltration, decreased inflammatory cytokines	[105, 189, 194]
	Mitochondrial ROS	Direct neutralization of mitochondrial ROS	MitoQ, SS-31	Restored mitochondrial function, decreased inflammation	[112–117]
			Phycocyanobillin	Reduced airway inflammation, prevention of airway remodeling	[179–182]
	Thioredoxin-1	ROS scavenging and MIF inhibition	Recombinant human Trx1	Reduced eosinophil recruitment, decreased mucus production	[125–129]
			d-α-tocopheryl acetate	Decreased F2-isoprostanes, reduced IL3/IL4, increased IL12	[174–178]
	Aldose Reductase	Inhibition of inflammatory signaling	Zopolrestat, Fidarestat	Suppressed Th2 cytokines, reduced airway hyperresponsiveness	[132–137]
			2,3,5,4′-tetrahydroxystilbene-2-O-β-d-glucoside	Reduced IgE levels, decreased airway inflammation	[185–188]
Inflammatory Signaling	JAK-STAT	Inhibition of cytokine signaling	Tofacitinib, LAS194046 (inhaled)	Reduced Th2 inflammation, improved lung function	[138–142]
			Salidroside	Modulation of GATA3/T-bet, decreased eosinophil counts	[226–230]
	NFκB	Prevention of inflammatory gene expression	IMD-0354, Andrographolide	Decreased airway inflammation and remodeling	[143–147]
			Resveratrol, Formononetin, Allicin	Reduced inflammation, improved respiratory health	[194–198, 215–223]
	NLRP3 Inflammasome	Inhibition of inflammasome assembly	MCC950, Oridonin	Reduced IL1β/IL18 production, improved steroid sensitivity	[148–155]
			Formononetin	Inhibition of ESR1/NLRP3/Caspase-1 pathway	[215]
	TLR7	Agonism shifts immune response from Th2 to Th1	Imiquimod, Resiquimod	Decreased allergic inflammation, improved lung function	[160]
	TLR2/TLR4	Antagonism reduces pro-inflammatory signaling	OPN-305, TAK-242	Suppressed allergen-induced inflammation	[153, 156]
			Resveratrol, Tectorigenin	Inhibition of HMGB1/TLR4/NFκB pathway	[196, 224]
MAPK Pathway	p38 MAPK	Inhibition of inflammatory signaling	SB203580, BIRB-796	Reduced pro-inflammatory cytokine production	[119, 163–166]
	ERK1/2	Inhibition of cellular proliferation and remodeling	MEK inhibitors (PD0325901)	Decreased airway remodeling, reduced mucus production	[119]
	JNK	Inhibition of pro-inflammatory gene expression	SP600125, JNK-IN-8	Suppressed inflammatory response	[163–166]
			Formononetin, Salidroside	Inhibition of JNK pathways, reduced oxidative stress	[219, 230]
GPCRs	DP2 (CRTH2)	Antagonism of PGD2 receptor	Fevipiprant, Timapiprant	Reduced eosinophilic inflammation, improved lung function	[169, 170, 170]
	GPER	Agonism enhances anti-inflammatory responses	G-1	Decreased Th2 cytokines, increased IL10 production	[167, 168, 168]
Epigenetic Regulators	HDACs	Restoration of histone acetylation balance	Trichostatin A, Vorinostat	Improved corticosteroid sensitivity, reduced inflammation	[199–210]
	microRNAs	Regulation of gene expression	miRNA mimics/antagomirs	Targeted modulation of specific pathways	[198]
			Resveratrol	Regulation of miRNA-34a targeting FoxP3	[198]
Dual-Acting	Nrf2 + NFκB	Simultaneous activation of Nrf2 and inhibition of NFκB	Dimethyl fumarate	Enhanced anti-inflammatory and antioxidant effects	[105, 189]
			Quercetin, Tectorigenin	SIRT1/Nrf2/HO-1 activation and NFκB inhibition	[105, 189]
	PDE3/4	Dual inhibition increases cAMP	RPL554 (Ensifentrine)	Combined bronchodilator and anti-inflammatory effects	[194–198]
Other Targets	Th1/Th2 balance	Modulation of immune response	Naringenin, 2,3,5,4′-tetrahydroxystilbene-2-O-β-d-glucoside	Suppressed IL5, reduced AHR, decreased airway remodeling	[185–188, 188, 231, 233–235]
	Cytokine modulation	Regulation of pro-/anti-inflammatory cytokines	Farnesol	Reduced IL6/IL10 ratio, decreased TNFα, COX2 inhibition	[236, 237, 237]
	Airway smooth muscle	Bronchodilation	Naringenin	Smooth muscle relaxation, improved mucociliary clearance	[233, 234, 234]

## Engineered nanodelivery systems to enhance the pharmacological efficacy and bioavailability of naturally derived bioactive compounds

Recent advancements in nanocarrier have highlighted the therapeutic potential of natural bioactive compounds in asthma through the strategic application of engineered nanoparticle delivery systems designed to improve the delivery efficiency and effectiveness of bioactive substances. Inhalable tea polyphenol-loaded poly-lactic-co-glycolic acid (PLGA) nanoparticles coated with platelet membranes have shown attenuation of asthmatic inflammation by enhancing biocompatibility, safety profiles, and retention in inflamed lungs [238]. Similarly, β-glucan-based nanoparticles mitigate acute asthma in mice by suppressing ferroptosis and DNA damage, and enhancing cytoprotection and immune modulation function [239]. Hydrophobic compound curcumin has been formulated into nanoparticles and nanocrystals to improve delivery efficiency and pulmonary bioactivity. Formulated curcumin significantly inhibits airway remodeling and inflammation through suppressing the proliferation, migration, and infiltration of airway smooth muscle cells and inflammatory mediator production [203, 204]. Phytochemicals, such as quercetin, naringenin, and baicalein, encapsulated into nanocarriers including solid lipid nanoparticles, chitosan nanoparticles, and hybrid microspheres, have shown significant enhancement in lung delivery efficiency and suppression of allergic airway pathology [240–242]. Additionally, self-assembling nanogels composed of binary small herbal molecules have been leveraged for antiviral synergy against respiratory syncytial virus [243]. Similarly, berberine- and magnolol-loaded monoolein- and PLGA-based nanoparticles effectively modulated epithelial remodeling and allergic responses [244, 245]. Collectively, these studies underscore the critical role of nanoscale delivery platforms in enhancing the pharmacokinetic profiles, lung bioavailability, and immunomodulatory efficacy of naturally derived bioactive compounds for the treatment and prevention of asthma.

## Traditional Chinese medicine formulations as potential regulators of oxidative stress and inflammation

Traditional Chinese Medicine (TCM) formulations show significant potential as asthma treatments by simultaneously targeting oxidative stress and inflammation. Synergic effect of the combined therapeutic impact of multiple herbs exceeds the sum of individual contributions through complementary actions across different pathways, enhanced bioavailability, and balanced therapeutic effects that provide comprehensive protection against asthma pathology. Innovative approaches combining conventional treatments with TCM may offer superior therapeutic outcomes by addressing the limitations of single-agent therapies. Table 5 provides a comprehensive summary of the TCM formulations and their mechanisms in regulating oxidative stress and inflammation in asthma treatment.

**Table 5:** Traditional Chinese medicine formulations for asthma treatment

Formula	Key components	Main mechanisms	Synergistic effects	Clinical/experimental findings	References
Pingchuan Formula	Traditional Chinese herbal prescription	• Inhibits PI3K/AKT/NFκB signaling • Neutralizes ROS • Enhances antioxidant defense systems • Modulates gut microbiota	• Simultaneous targeting of inflammation and oxidative stress • Combined effects on gut-lung axis	• Decreased levels of TNFα, IL4, IL6, IL13 • Reduced inflammatory infiltration • Improved gut-lung axis function • Restored oxidant-antioxidant balance	[246]
Ze-Qi-Tang (ZQT)	Nine herbs with *Euphorbia helioscopia* (Ze Qi) as main ingredient	• Inhibits PI3K/AKT/NFκB pathway • ROS scavenging • Enhances endogenous antioxidant enzymes • Reduces MMP9 and MMP3 expression	• Complementary actions across inflammatory and oxidative pathways • Enhanced bioavailability of active compounds	• Decreased pro-inflammatory cytokine production • Reduced nuclear translocation of NFκB • Repressed airway smooth muscle cell proliferation	[247]
Combined Extracts of Epimedii Folium and Ligustri Lucidi Fructus (EEL)	*Epimedii Folium* (EF) and *Ligustri Lucidi Fructus* (LLF)	• Modulates TGFβ/Smads pathway • Neutralizes ROS • Enhances Nrf2 expression • Inhibits inflammatory cell infiltration	• Remarkable synergistic effects in combating both inflammation and oxidative stress • Rich array of complementary antioxidant compounds	• Reduced IL4, IL5, and IgE levels • Decreased goblet cell hyperplasia • Reduced collagen deposition • Decreased eosinophil infiltration	[248]
Budesonide with EEL Combination (Bud&EEL)	Budesonide plus EEL	• Promotes apoptosis of inflammatory cells • Inhibits excessive autophagy • Regulates pro/anti-apoptotic proteins • Modulates TGFβ/Smads pathway	• Synergistically targets both inflammation and oxidative stress • Addresses limitations of conventional glucocorticoid therapy alone • Enhanced regulation of TGFβ/Smads pathway	• Superior reduction of oxidative damage markers • Decreased airway wall thickening • Reduced smooth muscle layer thickness • More effective than either component alone	[249]
Anti-Asthma Herbal Medicine Intervention (ASHMI)	Lingzhi (*Ganoderma lucidum*), Kushen (*Sophora flavescens*), Gancao (*Glycyrrhiza uralensis*)	• Inhibits Th2 cytokines (IL4, IL5) production • Suppresses eotaxin-1 secretion	• Interaction indices below 1 for inhibition of IL4, IL5, and eotaxin-1 • Combined effect greater than the sum of individual herbs	• Increased lung function • Reduced symptom scores and β2-agonist use • Similar efficacy to prednisone without adverse effects • First herbal medicine to receive FDA approval for phase I/II clinical trials	[250]

### Pingchuan formula

Pingchuan formula is a traditional Chinese herbal prescription with demonstrated efficacy in treating asthma through its potent anti-inflammatory and antioxidant properties. The formula primarily reduces airway inflammation by inhibiting the PI3K/Akt/NFκB signaling pathway, leading to decreased levels of pro-inflammatory cytokines TNFα, IL4, IL6, and IL13 [246]. Its rich content of flavonoids and polyphenols helps neutralize ROS and enhance endogenous antioxidant defense systems, restoring the oxidant-antioxidant balance disrupted in asthma. Additionally, Pingchuan formula modulates gut microbiota composition, increasing beneficial bacteria that produce anti-inflammatory short-chain fatty acids, thereby establishing a favorable gut environment that indirectly reduces systemic inflammation and oxidative stress through the gut-lung axis [246]. Research has shown that the water extract of the Pingchuan formula significantly ameliorates murine asthma by alleviating inflammatory infiltration, indicated by decreased levels of inflammatory cytokines in lung tissues. The main characteristics and synergistic effects of key TCM formulations for asthma treatment are summarized in Table 4.

### Ze-Qi-Tang formula

Ze-Qi-Tang (ZQT) is a traditional Chinese medicine preparation composed of nine herbs, with Euphorbia helioscopia (Ze Qi) as the main ingredient, demonstrating significant anti-inflammatory and antioxidant activities. ZQT exerts powerful anti-inflammatory effects through comprehensive inhibition of the PI3K/Akt/NFκB pathway, reducing the nuclear translocation of NFκB and decreasing the production of pro-inflammatory cytokines in the airways [247]. The formula contains multiple components with direct ROS scavenging abilities that neutralize free radicals while enhancing the expression and activity of endogenous antioxidant enzymes such as superoxide dismutase, catalase, and glutathione peroxidase. ZQT also targets inflammatory mediators by reducing the expression of MMP9 and MMP3, directly addressing the inflammatory component of airway remodeling in chronic asthma [247]. In experimental studies, ZQT treatment notably repressed cell viability and proliferation of airway smooth muscle cells induced by PDGF-BB, which plays a critical role in airway remodeling.

### Combined extracts of *Epimedii Folium* and *Ligustri Lucidi Fructus*

The combination of *Epimedii Folium* (EF) and *Ligustri Lucidi Fructus* (LLF) demonstrates remarkable synergistic effects in combating both inflammation and oxidative stress in asthma. The combined extracts (EEL) exert potent anti-inflammatory effects by reducing the levels of IL4, IL5, and IgE while containing a rich array of antioxidant compounds, particularly flavonoids and iridoid glycosides, that neutralize ROS and enhance Nrf2 expression [248]. EEL effectively modulates the TGFβ/Smads pathway, which links oxidative stress to inflammatory responses and tissue remodeling, by reducing the expression of TGFβ2 and Smad2 while increasing inhibitory Smad7 [248]. This interrupts the cycle where oxidative stress triggers inflammation, which in turn generates more oxidative stress. EEL also inhibits the infiltration of inflammatory cells, particularly eosinophils, which are major sources of ROS in asthmatic airways, thereby decreasing both local inflammation and oxidative burden [248]. Studies have shown that EEL significantly reduces goblet cell hyperplasia and collagen deposition in the airways of asthmatic rats.

### Budesonide with EEL combination

The co-administration of budesonide with EEL represents an innovative approach that synergistically targets both inflammation and oxidative stress, addressing the limitations of conventional glucocorticoid therapy alone. This combination functions through sophisticated regulation of cellular processes, promoting apoptosis of inflammatory cells while inhibiting excessive autophagy, and uniquely regulating the balance of pro-apoptotic and anti-apoptotic proteins [249]. Budesonide with EEL modulates the expression of α-SMA and Ki-67, markers of airway smooth muscle cell proliferation, and comprehensively affects the TGFβ/Smads pathway by decreasing TGFβ1 and Smad3 while increasing Smad7, more effectively than either component alone [249]. Studies show that Budesonide with EEL has superior effects on reducing markers of oxidative damage in lung tissues compared to budesonide alone, explaining its enhanced ability to reduce collagen deposition and goblet cell hyperplasia, processes driven by both inflammation and oxidative stress [249]. Research has demonstrated that Budesonide with EEL significantly decreased airway wall thickening and smooth muscle layer thickness, which are key features of airway remodeling in chronic asthma.

### Anti-asthma herbal medicine intervention (ASHMI)

ASHMI is a simplified Chinese herbal formula consisting of three herbs, including Lingzhi (*Ganoderma lucidum*), Kushen (*Sophora flavescens*), and Gancao (*Glycyrrhiza uralensis*). ASHMI works through multiple mechanisms, such as inhibiting Th2 cytokines (IL4 and IL5) production by memory Th2 cells, suppressing eotaxin-1 secretion by human lung fibroblasts, and demonstrating synergistic effects among its constituents as proven by interaction indices below 1 for anti-inflammatory activities [250]. The formula has shown efficacy in clinical studies, increasing lung function while reducing symptom scores and β2-agonist use similar to prednisone but without adverse effects on adrenal function or immune suppression [250]. ASHMI became the first herbal medicine to receive FDA approval for phase I/II clinical trials as an investigational new drug (IND No. 71526) for asthma treatment.

## Conclusions

The therapeutic landscape for asthma management is evolving beyond the conventional pharmacological approaches toward integrative strategies that incorporate naturally derived bioactive compounds. This paradigm shift is particularly relevant for addressing treatment-resistant phenotypes and mitigating long-term adverse effects associated with traditional medications. The complementary mechanisms underlying the effects of natural compounds—operating at the intersection of oxidative and inflammatory pathways—present unique opportunities for therapeutic innovation.

Despite the promising preclinical evidence regarding the efficacy of these natural compounds, translating the findings into clinical practice remains limited by several challenges, including issues related to bioavailability, optimal dosing, standardization of extracts, and the need for well-designed clinical trials to validate the efficacy and safety of the therapies in diverse asthma phenotypes. Future research should be focused on optimizing delivery systems to enhance bioavailability, developing combination therapies that leverage synergistic effects between natural compounds, and identifying specific asthma endotypes that may benefit most from these interventions.

In conclusion, naturally derived bioactive compounds represent a valuable frontier in asthma research, offering mechanistically distinct approaches to addressing the underlying pathophysiology of the disease. Continued investigation of these compounds may lead to the development of novel therapeutic strategies that can effectively manage symptoms, slow disease progression, and improve the quality of life of patients with asthma, particularly those who respond inadequately to conventional treatments.

## Data Availability

No data was used for the research described in the article.

## Abbreviations

AHR: Airway hyperresponsiveness
AP-1: Activator protein 1
AR: Aldose reductase
CAT: Catalase
CI: Confidence interval
COPD: Chronic obstructive pulmonary disease
CPC: C-phycocyanin
DALYs: Disability-adjusted life years
DC: Dendritic cell
ERK: Extracellular signal-regulated kinase
FEV_1_: Forced expiratory volume in one second
FVC: Forced vital capacity
GATA3: GATA binding protein 3
GPER: G-protein-coupled estrogen receptor
GPCR: G-protein-coupled receptor
GSH: Glutathione
HDM: House dust mite
HO-1: Heme oxygenase1
IFNγ: Interferon gamma
IgE: Immunoglobulin E
IL: Interleukin
ILC2: Innate type 2 lymphoid cell
JAK: Janus kinase
JNK: C-Jun N-terminal kinase
Keap1: Kelch-like ECH-associated protein 1
MAPK: Mitogen-activated protein kinase
MDA: Malondialdehyde
MIF: Macrophage migration inhibitory factor
MitoQ: MitoQuinone
MMP: Matrix metalloproteinases
NFκB: Nuclear factor kappa B
NLRP3: NOD-like receptor family, pyrin domain containing 3
NLR: NOD-like receptor
Nrf2: Nuclear factor erythroid 2-related factor 2
OR: Odds ratio
OVA: Ovalbumin
PGD2: Prostaglandin D2
PI3K: Phosphoinositide 3-kinase
PM: Particulate matter
ROS: Reactive oxygen species
S1P: Sphingosine-1-phosphate
scRNA-seq: Single-cell RNA sequencing
SIRT1: Sirtuin 1
SOD: Superoxide dismutase
SSRA: Severe steroid-resistant asthma
STAT: Signal transducer and activator of transcription
TGFβ: Transforming growth factor beta
Th: T helper
TLR: Toll-like receptor
TNFα: Tumor necrosis factor alpha
Treg: Regulatory T cell
Trx1: Thioredoxin-1
TSG: 2,3,5,4′-Tetrahydroxystilbene-2-O-β-d-glucoside
TWEAK: TNF-related weak inducer of apoptosis
XBP-1: X-box binding protein 1
